# Participant recruitment and retention in a pilot program to prevent weight gain in low-income overweight and obese mothers

**DOI:** 10.1186/1471-2458-9-424

**Published:** 2009-11-21

**Authors:** Mei-Wei Chang, Roger Brown, Susan Nitzke

**Affiliations:** 1 College of Nursing, Michigan State University, East Lansing, Michigan, USA; 2 School of Nursing and Department of Family Medicine, University of Wisconsin-Madison, Madison, Wisconsin, USA; 3 Department of Nutritional Sciences, University of Wisconsin-Madison, Madison, Wisconsin, USA

## Abstract

**Background:**

Recruitment and retention are key functions for programs promoting nutrition and other lifestyle behavioral changes in low-income populations. This paper describes strategies for recruitment and retention and presents predictors of early (two-month post intervention) and late (eight-month post intervention) dropout (non retention) and overall retention among young, low-income overweight and obese mothers participating in a community-based randomized pilot trial called *Mothers In Motion*.

**Methods:**

Low-income overweight and obese African American and white mothers ages 18 to 34 were recruited from the Special Supplemental Nutrition Program for Women, Infants, and Children in southern Michigan. Participants (n = 129) were randomly assigned to an intervention (n = 64) or control (n = 65) group according to a stratification procedure to equalize representation in two racial groups (African American and white) and three body mass index categories (25.0-29.9 kg/m^2^, 30.0-34.9 kg/m^2^, and 35.0-39.9 kg/m^2^). The 10-week theory-based culturally sensitive intervention focused on healthy eating, physical activity, and stress management messages that were delivered via an interactive DVD and reinforced by five peer-support group teleconferences. Forward stepwise multiple logistic regression was performed to examine whether dietary fat, fruit and vegetable intake behaviors, physical activity, perceived stress, positive and negative affect, depression, and race predicted dropout as data were collected two-month and eight-month after the active intervention phase.

**Results:**

Trained personnel were successful in recruiting subjects. Increased level of depression was a predictor of early dropout (odds ratio = 1.04; 95% CI = 1.00, 1.08; p = 0.03). Greater stress predicted late dropout (odds ratio = 0.20; 95% CI = 0.00, 0.37; p = 0.01). Dietary fat, fruit, and vegetable intake behaviors, physical activity, positive and negative affect, and race were not associated with either early or late dropout. Less negative affect was a marginal predictor of participant retention (odds ratio = 0.57; 95% CI = 0.31, 1.03; p = 0.06).

**Conclusion:**

Dropout rates in this study were higher for participants who reported higher levels of depression and stress.

**Trial registration:**

Current Controlled Trials NCT00944060

## Background

Recruitment and retention are important determinants of success for programs to improve nutrition and lifestyle behaviors, especially when participants are from low-income and racial/ethnic minority populations [1,2]. Recently, weight loss interventions that focused on physical activity and/or dietary intake behaviors among middle-aged overweight and obese adults have shown less than optimal retention rate: 47.0%-74.7% at six months [3-6] and 42.5%-70% [3,7-11] at one-year follow up.

Factors contributing to low retention (high dropout) rates among middle-aged overweight and obese women who participated in weight loss interventions have been reported. These predictors included poor eating behaviors [7,12,13], less physical activity [7,12], greater stress, higher emotional disturbance [12], more depression [7,12,13], and non-white race/ethnicity [14]. This study examines potential predictors of dropout in a *Mothers In Motion *(*MIM*) program to prevent weight gain among young, low-income overweight and obese mothers. The *MIM *intervention messages were delivered to the young mothers (ages 18-34) through an interactive DVD (IDVD) and a series of peer support group teleconferences (PSGTs). The *MIM*'s innovative approach was refined in a pilot study (*P-MIM*) via a partnership with Michigan State University and Michigan's Special Supplemental Nutrition Program for Women, Infants, and Children (WIC).

The objectives of the *P-MIM *were to 1) evaluate the intervention in terms of treatment fidelity and acceptability, 2) identify successful strategies for participant recruitment and maintaining active participation, and 3) collect and analyze preliminary indicators of *P-MIM*'s effect on dietary intake, physical activity, stress, affect, and body weight, compared to a control group. This paper describes effective strategies for recruitment and retention in the *P-MIM *(objective 2) and examines whether dietary fat, fruit, and vegetable intake behaviors, physical activity, perceived stress, positive and negative affect, depression, and race are significant predictors of early (two-month post intervention or six months from baseline) and late (eight-month post intervention or one-year from baseline) dropout and overall retention among study participants. We also present recommendations to improve recruitment and retention of low-income participants in studies with similar populations and intervention approaches.

## Methods

### Settings

Participants were recruited from three WIC programs in southern Michigan. WIC is a federally funded program that provides nutrition consultation and other services to low-income pregnant/breastfeeding women and young children (< 5 years).

### Procedure

Every woman coming to the collaborating WIC clinics during the data collection dates (June-July, 2007) was personally invited by trained recruiters to provide demographic information via a self-administered questionnaire. Then, women were invited to be screened except those who were obviously pregnant or older than 34 years. The screening survey addressed inclusion and exclusion criteria. Our enrollment process aimed for equal numbers of participants in two racial groups (African American and white) and three body mass index (BMI) categories (25.0-29.9 kg/m^2^, 30-34.9 kg/m^2^, and 35.0-39.9 kg/m^2^).

#### Inclusion criteria

Non-pregnant African American and white women between 18 and 34 years old who understood and spoke English and had a measured BMI between 25.0 and 39.9 kg/m^2 ^were eligible for this study. Additional inclusion criteria included having a youngest child between 6 weeks and 3.5 years of age enrolled in one of our three collaborating WIC programs, not planning to become pregnant or change WIC clinics during the study, providing accurate contact information, and agreeing to provide updates for contact information and pregnancy status, willingness to accept randomized participation assignments, agreeing to participate in the project for 1.5 years, and willingness to have blood glucose tested via finger stick. Exclusion criteria. Women with fasting blood glucose greater than 126 mg/dl or random (non-fasting) blood glucose greater than 200 mg/dl, self-reported type 1 or 2 diabetes or an eating disorder, or inability to walk more than one block without resting or shortness of breath were excluded. Eligible participants provided their individual telephone and address and a telephone number of one back-up contact (relative, friend, or neighbor). The study procedure was approved by the Institute of Review Board at Michigan State University. Consent forms included demographic data for randomization.

### Recruitment Strategies

We enhanced recruitment by emphasizing confidentiality and enlisting positive support from WIC personnel at the collaborating program sites. During recruitment, a DVD was used to help potential participants become familiar with the *MIM *study's materials and protocol, including requirements and incentives. Recruiters were Asian or white students and University staff who were trained to be culturally sensitive, speak clearly, and listen respectfully. They explained the study's purpose, requirements, confidentiality, flexible scheduling, benefits of participation (e.g., no-cost prevention of weight gain with strategies to mange stress), and incentives. They also emphasized that *P-MIM *was a collaborative effort between WIC and Michigan State University.

### Retention Strategies

During recruitment, we obtained two working phone numbers from each participant. Also, they were asked to provide the best time to reach for a 45-55 minute interview. Trained interviewers used a flexible schedule including nights/weekends, provided toll-free numbers for return calls, made repeated calls over a 4-week period if necessary to reach participants, and called the same assigned participants for interview throughout the project. A computerized tracking system kept confidential records of participants' responses and each attempted phone contact. We mailed easy-to-read reminders before and sent thank-you letters after each phone interview. The *MIM *logo, developed with input from WIC partners and the target audience, was used on all correspondence mailed materials for this study. To further maintain contact, we mailed birthday and holiday greeting cards to participants.

We provided a $5.00 incentive when participants contacted us via phone or a postcard with a change of address, phone number, or pregnancy status. We also provided incentives that reimbursed participants for their time and cell phone costs, e.g., $40 for completing a telephone interview and returning to the WIC clinic where they had been recruited to have their body weight measured. At the final data collection, a bonus incentive was provided: $10 for a completion of the final data collection and $20 if interviews were complete for all three time points of data collection.

### Stratified Randomization

Following the baseline telephone interview, participants were randomly assigned to an intervention (diet, physical activity, stress management, usual WIC care, n = 64) or a control group (usual WIC care and an option of receiving IDVD at the end of the study, n = 65) equalized for racial representation and three BMI categories.

### Intervention

This 10-week pilot intervention was developed following a community-based participatory research model. Its theory-based culturally sensitive educational messages were delivered via a series of five chapters in an IDVD (10-15 minutes/chapter) that were reinforced by five PSGTs (30 minutes/per session). The IDVD featured peers (overweight and obese WIC mothers) and the PSGTs were led by educators from three collaborating WIC programs in Michigan. The five chapter topics in the IDVD were stress management and avoiding eating foods for comfort, ways to be physically active with young children, grocery shopping and food label reading, meal planning, and food preparation. Each chapter included an interactive information presentation (1-2 minutes), culturally sensitive narratives (7-10 minutes), a section on setting personal goals (2-3 minutes), and three quiz questions (~20 seconds) to verify attention to the content.

A package of intervention materials with the *MIM *logo was sent via certified mail (signature required) to intervention participants' homes. The intervention package included one five-chapter IDVD, five weekly worksheets, five quizzes, five pamphlets, two postcards for reporting changes of address or phone number, and one postcard for pregnancy status notification.

#### Viewing IDVD at home

Intervention participants were asked to view a designated chapter in the IDVD every other week (10-15 minutes/chapter) for 10 weeks and to refrain from sharing the IDVD with other WIC mothers during the study. After viewing a designated chapter, they answered three quiz questions and wrote one or two short- and long-term goals on their weekly worksheets, and monitored their progress for seven days by circling whether their progress that day was 'not so great," "so so," or "great." Weekly worksheet and quiz questions were mailed to the study office.

#### PSGTs

Intervention participants were asked to call in to a scheduled PSGT the week after they viewed a designated chapter in the IDVD. A moderator and assistant moderator were on-line as participants called in to PSGTs. The moderator opened the group with an "ice-breaker" activity and introduced ground rules and specific subtopics that were consistent with contents presented in the previous week of IDVD at the beginning of each session. Participants were asked to share their personal goals, report problems with application, and encourage each other to make positive lifestyle behavioral changes. The moderator assisted in problem solving, identified barriers in behavior changes, and assessed participants' ability to apply learned cognitive skills to daily life. The assistant moderator took notes and kept track of who was online. The session closed with participants' reviews of lessons learned and possible new strategies for behavioral changes. The moderator also reminded participants of the following week's topics in the IDVD and procedures.

### Usual WIC care

Regardless of her group assignment, each participant received WIC nutrition education for approximately 20 minutes every six months during the re-certification appointment for her young child(ren).

### Measures

Survey data were collected by telephone interview and body weight was measured at collaborating WIC clinics at three time points: baseline, two-month post intervention (six-month follow up), and eight-month post intervention (one-year follow up).

#### Fat, fruit, and vegetable intake

The National Cancer Institute (NCI) 15-item Fat Screener and algorithm with established predictive validity was used to generate a fat intake score, with a higher score indicating a higher percent of calories from fat [15,16]. The NCI fruit and vegetable Short Assessment Form and algorithm with established predictive validity (19 items) were used to generate a score, with a higher score indicating a higher fruit and vegetable intake [17,18].

#### Physical activity

The Godin Leisure Time Exercise Questionnaire with established validity and reliability was used to measure moderate physical activity (6 items) [15]. Participants were asked how frequently and for how long they participated in moderate physical activity in the last seven days (i.e., walking, jogging, biking, aerobic exercise, dancing, and playing active activities with children). We multiplied METs (metabolic equivalent hours per week) for each activity [16] and summed six activities to a single score with a higher score indicating more physical activity.

#### Perceived stress

The Perceived Stress Scale (9 items) with established validity and reliability was used to measure stress. Responses to each item were rated on a 4-point scale ranging from 1 (rarely or never) to 5 (usually or always) [17]. Nine items were summed to create a score with a higher score indicating less stress.

#### Affect

The 18-item Positive Affect and Negative Affect Scale (PANAS) with established validity and reliability was used to measure affect, a prevalent and relevant outcome of stress [18,19]. Participants responded to a list of words that describe different feelings and emotions, e.g., happy, interested, strong (positive affect), stress, upset, and guilty (negative affect). Responses to each item were rated on a 5-point scale ranging from 1 (very slightly or not at all) to 5 (extremely) [20]. Eleven negative affect items were summed to create a score with a higher score indicating experience of less negative affect. Similarly, seven positive affect items were summed to create a score with a higher score indicating stronger positive affect.

#### Depression

The Center for Epidemiologic Studies Depression Scale (CES-D) with established validity and reliability was used to measure depression (20 items). Responses to each item were rated on a 4-point scale ranging from 0 (rarely or none of the time) to 3 (most or all of the time) [21]. Twenty items were summed to create a score with a higher score indicating more depression.

#### Height and weight

Height without shoes was measured to the nearest 0.1 cm using a wall-mounted stadiometer. Weight was measured to the nearest 0.1 kg on an electronic scale with the participants wearing light clothing and no shoes. These two measurements were done at the collaborating WIC clinics. BMI was calculated from weight (kg)/height (m^2^). Baseline height and weight were measured by trained recruiters and follow up body weight was measured by trained WIC staff members.

#### Qualitative evaluation

Procedures were evaluated via recruiters' log notes, focus groups with *P-MIM *intervention participants, and focus groups with WIC personnel. Of the 28 intervention participants who had completed phone interviews (two-month post intervention) and were invited to attend focus group discussions, 12 participants attended one of three focus groups. In addition, five focus groups were conducted with a total of 25 WIC personnel. A trained moderator used a semi-structured interview guide to lead the group discussions. A trained research assistant audio-taped the proceedings and took notes. At the end of each focus group discussion, the moderator verified the data collected by summarizing main points from the discussion and asking participants if any key ideas were missed. The group discussion took place in collaborating County Health Department meeting rooms. Participants signed consent forms before participation and the procedure was approved by Michigan State University Institute of Review Board.

### Data Analysis

#### Quantitative data analysis

Women who became pregnant (n = 5 at six-month follow up, n = 6 between six-month and one-year follow up) during the trial were excluded from analysis because of violation of inclusion criteria. T-test and Mann-Whitney tests were used to analyze continuous variables and chi-square was used for analysis of categorical variables. Forward stepwise multiple logistic regression was performed to identify predictors of early and late dropout and overall retention, all via NCSS software [22]. We defined dropouts as participants who did not complete either telephone interview or a body weight measure at one-year follow up. *Qualitative data analysis*. Common themes were identified from recruiters' log notes. Research assistants transcribed focus group audiotapes and incorporated the moderator's field notes. Two research assistants coded data independently to identify common themes with 96% inter-rater agreement.

## Results

### Sample Representation

**Figure 1:**
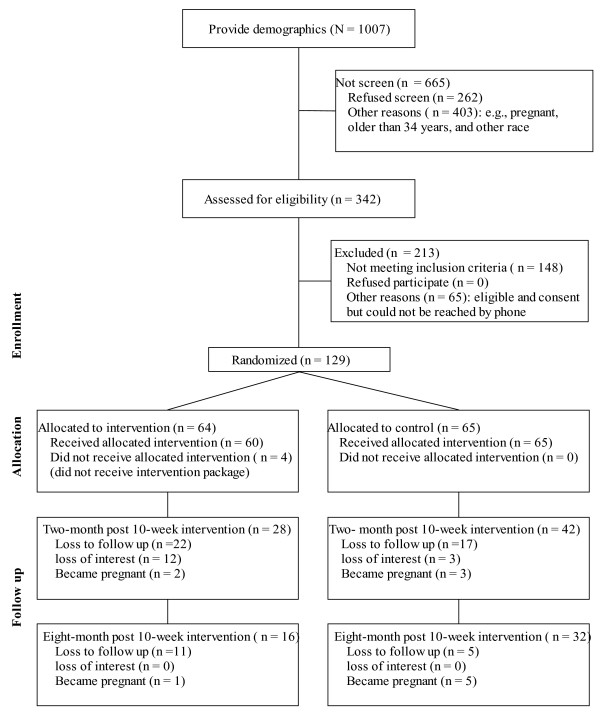
**Sample Representation and Retention: CONSORT Chart**.

All recruitment BMI/race sub-categories were filled by our trained recruiters in two months instead of planned four months. Figure 1 presents data on sample representation (CONSORT chart). Of women who were invited to be screened for the *P-MIM *study, 90% (n = 1007) provided demographic information and 342 (33.96%) completed the screening. The main reasons for not being screened were age > 34 years, being pregnant, and lack of interest. Of 342 women screened, 194 (56.7%) were eligible and consented. The main reasons for not being eligible were BMIs outside targeted ranges, race-BMI categories being full, and age > 34 years. Of 194 women, 129 (66.5%) were enrolled. The main reason for non-enrollment (n = 65) was being unreachable by phone.

Compared to those not in the trial (n = 878), a higher proportion of those in the trial (n = 129) had at least some college education (p < 0.05). Compared to women who were eligible but not enrolled (n = 65), those eligible and enrolled (n = 129) were 1.5 years older (p < 0.05) and were more likely to be former smokers (p < 0.05). No other differences were found in racial groups and BMI categories between these two groups.

### Demographics

Table 1 presents demographic comparison of retainers (n = 48) and dropouts (n = 70). Dropouts were an average of 1.7 years younger (p < 0.05) than participants who completed the study. There was a significant difference in education between these two groups (p < 0.05). However, there were no significant differences in postpartum status, BMI, race, smoking status, or employment status between these two groups.

**Table 1:** Demographic Comparison of Retainers and Dropouts (N = 118)

Demographics	Retainers (n = 48)		Dropouts (n = 70)		Statistical test	P-value
	**M**	**SD**	**M**	**SD**		

Age (years)	26.4	4.10	24.7	3.80	t(116) = 2.3	0.02

Postpartum (years)	1.31	0.83	1.03	0.81	t(116) = 1.8	0.07

Body mass index (kg/m^2^)	32.03	4.49	31.67	3.88	t(116) = 0.47	0.64

	**N**	**%**	**N**	**%**		

Race					χ^2 ^(1) = 0.462	0.57^a^

African American	23	47.92	38	54.29		

White	25	52.08	32	45.71		

Smoking status^1^					χ^2 ^(2) = 0.94	0.65^a^

Never smoked	21	43.75	36	52.17		

Smoke but quit	16	33.33	18	26.09		

Smokers	11	22.92	15	21.74		

Education					χ^2 ^(3) = 13.78	0.00^a^

Some high school	6	12.50	15	21.43		

High school	25	52.08	31	44.29		

Some college or technical school	6	12.50	21	30.00		

College degree or higher	11	22.92	3	4.29		

Employment Status					χ^2 ^(6) = 7.76	0.26^a^

Full time	11	22.92	16	22.86		

Part time	10	20.83	14	20.00		

Homemaker	15	31.25	9	12.86		

Self-employed	1	2.08	3	4.29		

Unemployed	7	14.58	17	24.29		

Students	3	6.25	9	12.86		

Other	1	2.08	2	2.86		

^1^N = 69 for dropouts. Retainers are defined as participants who completed either telephone interview or a body weight measure at one-year follow up. ^a^Exact probabilities

### Change of Address and Phone Numbers

Of 129 women enrolled in the study, 48 (37.2%) participants notified us for a change of address (n = 16, 12.4%), phone number (n = 30, 23.3%), or both (n = 2, 1.6%) during the one-year project. Seventeen participants (35.42%) notified us for a change of contact information two or more times. The community advisory group explained that phone disconnections and changed numbers were frequent because of different "pay as you go" (pre-paid) phone plans as well as missed/late payments on traditional phone bills.

### Retention Rates

Figure 1 presents sample retention. Of 118 participants, 70 (59.3%, six months follow up) and 48 (40.7%, one-year follow up) completed telephone interviews and 58 (49.2%, six months follow up) and 39 (33.0%, one-year follow up) returned to the WIC clinics for a body weight measure. The main reasons for dropout were loss to follow up (54/118, 45.8%; mainly due to disconnected phones) and self-reported loss of interest (16/118, 13.6%).

### Predictors of Early and Late Dropout and Overall Retention

Table 2 presents scores for retainers and dropouts. No significant differences were found in lifestyle behaviors and psychosocial variables. Table 3 presents predictors of early and late dropout and overall retention. *Early dropout*. Forward stepwise multiple logistic regression showed that each unit score incremental increase of CES-D (depression scale) was associated with a participant being 1.04 times more likely to dropout (odds ratio = 1.04; 95% CI = 1.00, 1.08; p = 0.03). Dietary fat, fruit, and vegetable intake behaviors, physical activity, perceived stress, positive and negative affect, and race were not associated with early drop out. *Late dropout*. Greater stress was a predictor of late dropout (odds ratio per unit of change in the perceived stress score = 0.20; 95% CI = 0.00, 0.37; p = 0.01). Dietary fat, fruit, and vegetable intake behaviors, physical activity, positive and negative affect, depression, and race were not associated with late dropout. *Overall *retention. Less negative affect predicted retention at the end of the study (odds ratio per unit of change in the negative affect scale = 0.57; 95% CI = 0.31, 1.03; p = 0.06).

**Table 2:** Comparison of Psychosocial and Lifestyle Behavior Variables between the Retainers and Dropouts (N = 118)

Variables	Retainers (n = 48)		Dropouts (n = 70)		Statistical test	P-value
	**M**	**SD**	**M**	**SD**		

**^1^Fat intake behaviors (% of total calories from fat)**	**33.87**	**6.37**	**34.27**	**7.30**	**t(116) = -0.31**	**0.75**

Fruit and vegetable intake behaviors (cups/day)	4.69	3.79	4.21	3.15	Z(116) = 0.73	0.46

Physical activity (MET)	31.48	28.49	25.55	24.66	Z(116) = 1.22	0.22

Perceived stress	2.24	0.28	2.19	0.29	t(116) = 0.96	0.34

Positive affect	3.36	0.73	3.33	0.69	t(116) = 0.24	0.80

Negative affect	3.76	0.68	3.55	0.62	t(116) = 1.78	0.08

**^1^Depression**	**15.31**	**10.13**	**18.13**	**10.59**	**t(116) = -1.44**	**0.15**

^1^Lower scores are more favorable. T-test was applied for variables with normal distribution. Mann-Whitney test (Z-value) was applied for variables without normal distribution.

**Table 3:** Predictors of Early and Late Dropouts and Overall Retention

Variables	β	SE	Odds ratio	95% confidence interval	P-value
Early dropout (Two-month post intervention): N = 124^1^					

CES-D	0.04	0.02	1.04	1.00, 1.08	0.03

Perceived stress	0.85	0.68	2.35	0.62, 8.88	0.21

Late dropout (Eight-month post intervention): N = 70^2^					

Perceived stress	-3.72	1.39	0.20	0.00, 0.37	0.01

Positive affect	0.81	0.50	2.24	0.84, 5.97	0.10

Overall retention: N = 118^3^					

Negative affect	-0.56	0.31	0.57	0.31, 1.03	0.06

Physical activity	-0.01	0.01	0.99	0.98, 1.00	0.18

SE = standard error. Reference group = retention

^1^N = 124. Of 129 participants, 5 became pregnant after baseline data collection and were excluded from the analysis.

^2^N = 70. Using two-month post intervention data for analysis.

^3^N = 118. Of 129 participants, 11 became pregnant during the one-year study. These women were excluded from data analysis.

### Recruiters' Log Notes

By showing respect, establishing rapport, and providing incentives, we were able to recruit young, low-income overweight and obese mothers that are often considered "hard to reach." Baby-sitting was critical because WIC personnel and mothers perceived it as a sign of caring. In fact, some WIC mothers who had refused to participate during the initial invitation decided to participate after they saw the positive interaction between the recruiters and their children. We also found talking to each woman individually was more successful than recruiting two or more participants at the same time.

### Focus Group Discussions for Recruitment/Screening Procedure and Retention

#### Recruitment/screening procedure

The *P*-*MIM *intervention participants said that the IDVD was about having a happy and healthy family so that the key message to recruit their peers should be "happy and healthy family" rather than "prevention of weight gain." *Retention*. Intervention participants. Lack of understanding study requirements and incentives affected retention rate. Mothers stated that they could not remember the study requirements and incentives after signing a consent form because of all the distractions at WIC clinics. They suggested providing a flyer with key study requirements and incentives for participants to take home and making reminders at the end of each telephone interview. WIC personnel. To increase retention rate, WIC personnel suggested having newly recruited study participants return to their WIC clinics where they were recruited to pick up the first study package as a condition of enrollment. Potential participants who return for this first step are more likely to follow through and actively participate throughout the study.

## Discussion

Researchers have reported the importance of matching racial background of recruiters and the target audience to enhance recruitment [23]. Our white and Asian recruiters were able to recruit African Americans and whites with nearly equal success, suggesting that training for cultural sensitivity can help overcome some potential recruitment problems.

Our findings verify findings of previous studies that reported difficulties in maintaining participation in longitudinal studies when phone numbers and addresses are not stable [1,24]. Therefore, it is critically important to include a provision for updating contact information in planned incentives for such studies.

Our retention rate at six-month follow up was 59.3%. Other researchers have reported six-month follow up retention rate of 47% for a community-based weight management program for overweight and obese adults [6] and 73% for a primary care weight management intervention for low-income African-American women aged 18 to 65 years old [5]. The potential for retention failures was relatively high in our study because we enrolled participants whose characteristics have previously been associated with lower retention: non-white, young (< 30 years) [14], healthy [6], and parents [14]; high rates of becoming pregnant during the study [14,25];. living with children [6,14]; having children older than 7 months [26], having low education [6,14]; low income [14,24]; and frequently changing to a different WIC clinic [24]. Findings of the current study demonstrate that extraordinary procedures (see recommendations for future studies) may be necessary in studies with these potential barriers to extended participation.

Consistent with previous studies [7,12,13], we found that participants who reported being more depressed were more likely than those who reported being less depressed to dropout at six-month follow up. Also, participants who reported greater stress were more likely than those who reported less stress to dropout at one-year follow up. Contradictory to previous studies [7,12,13], we did not find associations between the dropout and dietary fat, fruit and vegetable intake behaviors, physical activity, positive and negative affect, and race. We found that WIC mothers who reported experiencing less negative affect were more likely to stay in this study than those who reported experiencing more negative affect. WIC mothers tend to be highly mobile and relocation has been reported to exacerbate stress for families [27]. Additionally, a worsening recession and the closing of several local automobile plants at the time of this study may have further decreased family incomes, exacerbated stress levels, and increased the tendency to move frequently.

Presenting a program to potential participants during the recruitment phase needs a careful planning. For example, we found that some overweight and obese mothers were not interested in a program to prevent weight gain because of their previous failures with weight loss programs. Some women were still looking for quick fixes instead of long-term lifestyle behavioral changes and others felt their excess weight was impossible to change, due to genetically determined tendencies [19].

There are limitations to this study. We are unable to identify which specific recruitment or retention strategies were more or less effective than others because we implemented numerous strategies simultaneously. This study was conducted with a targeted sample of overweight and obese African American and white mothers aged 18 to 34 years, which limits the generalizability of our findings to other groups such as normal weight or morbidly obese mothers or other low-income populations. Also, interpretation of the findings needs to be cautious because of a relatively large dropout rate.

Based on the results of the *P-MIM *and our experience of working with WIC mothers, we propose the following strategies for researchers working with low-income populations to potentially improve recruitment and retention.

1. Emphasize positive shared goals such as "healthy and happy family." This is consistent with the lessons reported by the U.S. Department of Agriculture's Food and Nutrition Service when developing a set of lessons for low-income mothers of preschoolers [28].

2. Consider a sequential screening that has been well documented in LOOK AHEAD [29], Women's Health Initiative [30], and other studies [31,32] to improve recruitment and retention. At the first screening, give eligible participants a culturally tailored easy-to-read flyer [33,34] that outlines study goals, expectations, and incentives as presented in the consent form to avoid not understanding or forgetting study requirements. Ask eligible participants to read the flyer followed by a brief interview (1-2 minutes) conducted by a trained recruiter to assess potential participants' interest and commitment and to determine if the study goals and expectations are a good fit. Ask eligible participants to consent to participate only if they can verbalize understanding of the study requirements and potential barriers to participation. In later sessions, assign one of the key study requirements (e.g., keep a 2-day food diary, return to a study site for physiological measurements) for participants to complete. Enroll participants only if they complete a key initial study requirement.

3. Consider training peers to serve as recruiters. Researchers have used peers to successfully recruit and retain minority participants [34-37].

4. Plan multiple ways to maintain contact with highly mobile participants. Require participants to provide three phone numbers with at least two having different addresses [38]. Provide a small incentive to encourage participants to update their address, three phone numbers, and pregnancy status monthly via phone or a postcard. Also, send a greeting card every month to maintain continuous contact with participants [39].

5. Offer interventions at times and locations that are convenient for study participants.

## Conclusion

We implemented a range of strategies to successfully recruit young, low-income overweight and obese African American and white mothers. Retention rates were less than ideal. It is likely that dropout rates were exacerbated by population characteristics and unusual economic hardships at recruitment sites. Addressing depression and emphasizing stress management are recommended to maximize retention in future studies of this nature.

## Competing interests

The authors declare that they have no competing interests.

## Authors' contributions

MC contributed to the design, development of intervention materials, data collection coordination and interpretation and development of the initial draft of the manuscript. RB participated in the design of the study, performed statistical analysis, interpreted findings, and participated in manuscript writing. SN contributed to the design, development of intervention materials, data interpretation, and manuscript writing. All authors read and approved the final manuscript.

## Pre-publication history

The pre-publication history for this paper can be accessed here:

http://www.biomedcentral.com/1471-2458/9/424/prepub

## References

[B1] Blumenthal DS Sung J Coates R Williams J Liff J Recruitment and retention of subjects for a longitudinal cancer prevention study in an inner-city black community Health Serv Res 1995 30 1 Pt 2 197 205 7721592 PMC1070049

[B2] Havas S Anliker J Greenberg D Block G Block T Blik C Langenberg P DiClemente C Final results of the Maryland WIC Food for Life program Prev Med 2003 37 5 406 416 10.1016/S0091-7435(03)00160-9 14572425

[B3] McManus K Antinoro L Sacks F A randomized controlled trial of a moderate-fat, low-energy diet compared with a low fat, low-energy diet for weight loss in overweight adults Int J Obes Relat Metab Disord 2001 25 10 1503 1511 10.1038/sj.ijo.0801796 11673773

[B4] Tate DF Wing RR Winett RA Using Internet technology to deliver a behavioral weight loss program JAMA 2001 285 9 1172 1177 10.1001/jama.285.9.1172 11231746

[B5] Davis Martin P Rhode PC Dutton GR Redmann SM Ryan DH Brantley PJ A primary care weight management intervention for low-income African-American women Obesity (Silver Spring) 2006 14 8 1412 1420 10.1038/oby.2006.160 16988084

[B6] Graffagnino CL Falko JM La Londe M Schaumburg J Hyek MF Shaffer LE Snow R Caulin-Glaser T Effect of a community-based weight management program on weight loss and cardiovascular disease risk factors Obes Res 2006 14 2 280 288 10.1038/oby.2006.36 16571854

[B7] Katzer L Bradshaw AJ Horwath CC Gray AR Obrien S Joyce J Evaluation of a "nondieting" stress reduction program for overweight women: A randomized trial Am J Health Promot 2008 22 4 264 274 18421891 10.4278/060728113R1.1

[B8] Dansinger ML Gleason JA Griffith JL Selker HP Schaefer EJ Comparison of the Atkins, Ornish, Weight Watchers, and Zone diets for weight loss and heart disease risk reduction: A randomized trial JAMA 2005 293 1 43 53 10.1001/jama.293.1.43 15632335

[B9] Martin PD Dutton GR Rhode PC Horswell RL Ryan DH Brantley PJ Weight Loss Maintenance Following a Primary Care Intervention for Low-income Minority Women Obesity (Silver Spring) 2008 16 11 2462 2467 10.1038/oby.2008.399 18787526 PMC2753427

[B10] Dalle Grave R Calugi S Molinari E Petroni ML Bondi M Compare A Marchesini G Weight loss expectations in obese patients and treatment attrition: An observational multicenter study Obes Res 2005 13 11 1961 1969 10.1038/oby.2005.241 16339128

[B11] Jeffery RW Sherwood NE Brelje K Pronk NP Boyle R Boucher JL Hase K Mail and phone interventions for weight loss in a managed-care setting: Weigh-To-Be one-year outcomes Int J Obes Relat Metab Disord 2003 27 12 1584 1592 10.1038/sj.ijo.0802473 14517547

[B12] Yass-Reed EM Barry NJ Dacey CM Examination of pretreatment predictors of attrition in a VLCD and behavior therapy weight-loss program Addict Behav 1993 18 4 431 435 10.1016/0306-4603(93)90060-M 8213297

[B13] Teixeira PJ Going SB Houtkooper LB Cussler EC Metcalfe LL Blew RM Sardinha LB Lohman TG Pretreatment predictors of attrition and successful weight management in women Int J Obes Relat Metab Disord 2004 28 9 1124 1133 10.1038/sj.ijo.0802727 15263921

[B14] Lohse B Stotts JL Bagdonis J Income sub stratification within a low income sample denotes dropout and completion patterns in nutrition education intervention for young adults The FASEB Journal 2006 20 5 A1312

[B15] Godin G Godin leisure-time exercise questionnaire Med Sci Sports Exerc 1997 29 6s S36

[B16] Ainsworth BE Haskell WL Leon AS Jacobs DRJ Montoye HJ Sallis JF Paffenbarger RSJ Compendium of physical activities: classification of energy costs of human physical activities Med Sci Sports Exerc 1993 25 1 71 80 10.1249/00005768-199301000-00011 8292105

[B17] Cohen S Kamarck T Mermelstein R A global measure of perceived stress J Health Soc Behav 1983 24 4 385 396 10.2307/2136404 6668417

[B18] Stone AA Cohen S, Kessler RC, Gordon LU Measurement of affective response Measuring Stress: A Guide for health and Social Scientists 1995 New York, NY: Oxford University Press 148 174

[B19] Chang M Nitzke S Guilford E Adair C Hazard D Motivators and barriers to healthful eating and physical activity among low-income overweight and obese mothers J Am Diet Assoc 2008 108 6 1023 1028 10.1016/j.jada.2008.03.004 18502238

[B20] Watson D Clark LA Tellegen A Development and validation of brief measures of positive and negative affect: the PANAS scales J Pers Soc Psychol 1988 54 6 1063 1070 10.1037/0022-3514.54.6.1063 3397865

[B21] Radloff LS The CES-D scale: A self-report depression, scale for research in the general population Applied Psychological Measurement 1977 1 35 385 401 10.1177/014662167700100306

[B22] Hintze J NCSS 2007 Kaysville, Utah: NCSS, LLC

[B23] Gavaler JS Bonharn-Leyba M Castro CA Harman SE The Oklahoma Postmenopausal Women's Health Study: Recruitment and Characteristics of American Indian, Asian, Black, Hispanic, and Caucasian Women Alcoholism: Clinical and Experimental Research 1999 23 2 220 223 10.1111/j.1530-0277.1999.tb04103.x 10069549

[B24] Damron D Langenberg P Anliker J Ballesteros M Feldman R Havas S Factors associated with attendance in a voluntary nutrition education program Am J Health Promot 1999 13 5 268 275 10538640 10.4278/0890-1171-13.5.268

[B25] Links of WIC Resources for Providers of WIC Services http://www.michigan.gov/documents/mdch/2006.state.pnss.tables_215430_7.pdf

[B26] Hammad TA Havas S Damron D Langenberg P Withdrawal rates for infants and children participating in WIC in Maryland J Am Diet Assoc 1997 97 8 893 895 10.1016/S0002-8223(97)00219-8 9259714

[B27] Berger PS Powell J Cook AS The relation of economic factors to perceived stress in mobile families Lifestyles 1988 9 4 297 313 10.1007/BF00986748

[B28] FNS Launches new initiative to help families make healthier choices http://www.fns.usda.gov/cga/PressReleases/2007/FNS-0001.htm

[B29] Ryan DH Espeland MA Foster GD Haffner SM Hubbard VS Johnson KC Kahn SE Knowler WC Yanovski SZ Look AHEAD (Action for Health in Diabetes): Design and methods for a clinical trial of weight loss for the prevention of cardiovascular disease in type 2 diabetes Control Clin Trials 2003 24 5 610 628 10.1016/S0197-2456(03)00064-3 14500058

[B30] Fouad MN Corbie-Smith G Curb D Howard BV Mouton C Simon M Talavera G Thompson J Wang CY White C Special populations recruitment for the Women's Health Initiative: successes and limitations Control Clin Trials 2004 25 4 335 352 10.1016/j.cct.2004.03.005 15296809

[B31] Levkoff S Sanchez H Lessons learned about minority recruitment and retention from the Centers on Minority Aging and Health Promotion Gerontologist 2003 43 1 18 26 12604742 10.1093/geront/43.1.18

[B32] Zayas LH McKee MD Jankowski KR Adapting psychosocial intervention research to urban primary care environments: a case example Ann Fam Med 2004 2 5 504 508 10.1370/afm.108 15506589 PMC1466732

[B33] Kreuter MW Lukwago SN Bucholtz RD Clark EM Sanders-Thompson V Achieving cultural appropriateness in health promotion programs: targeted and tailored approaches Health Educ Behav 2003 30 2 133 146 10.1177/1090198102251021 12693519

[B34] Yancey AK Ortega AN Kumanyika SK Effective recruitment and retention of minority research participants Annu Rev Public Health 2006 27 1 28 10.1146/annurev.publhealth.27.021405.102113 16533107

[B35] Resnicow K Campbell MK Carr C McCarty F Wang T Periasamy S Rahotep S Doyle C Williams A Stables G Body and soul. A dietary intervention conducted through African-American churches Am J Prev Med 2004 27 2 97 105 10.1016/j.amepre.2004.04.009 15261895

[B36] Arean PA Alvidrez J Nery R Estes C Linkins K Recruitment and retention of older minorities in mental health services research Gerontologist 2003 43 1 36 44 12604744 10.1093/geront/43.1.36

[B37] Larkey LK Staten LK Ritenbaugh C Hall RA Buller DB Bassford T Altimari BR Recruitment of Hispanic women to the Women's Health Initiative. The case of Embajadoras in Arizona Control Clin Trials 2002 23 3 289 298 10.1016/S0197-2456(02)00190-3 12057880

[B38] Robinson KA Dennison CR Wayman DM Pronovost PJ Needham DM Systematic review identifies number of strategies important for retaining study participants J Clin Epidemiol 2007 60 8 757 765 10.1016/j.jclinepi.2006.11.023 17606170 PMC1997303

[B39] Coday M Boutin-Foster C Goldman Sher T Tennant J Greaney ML Saunders SD Somes GW Strategies for retaining study participants in behavioral intervention trials: retention experiences of the NIH Behavior Change Consortium Ann Behav Med 2005 29 Suppl 55 65 10.1207/s15324796abm2902s_9 15921490

